# Western Iowa Veterinary Medical Association

**Published:** 1892-05

**Authors:** L. U. Shipley

**Affiliations:** Secretary


*Western Iowa Veterinary Medical Association.*—The fifth meeting of the Western Iowa Veterinary Medical Association met in the parlor of Burk's Hotel, Carroll, Ia., on Wednesday evening, March 23, 1892, pursuant to the call of the Secretary, and was called to order by President G. J. Gibson, after which, in a few well-chosen remarks, the President thanked the Association for the honor conferred upon him at the last meeting, during his absence, also expressing his gratification upon the growth of the Association. Upon roll call those present were: President G. J. Gibson, Vice-President G. C. Williams, Secretary L. U. Shipley, Professor W. B. Niles, of Ames, S. H. Johnston, G. A. Jonson, and Dr. Heck, of Harlan, as a visitor. The minutes of the previous meeting were read by the Secretary and approved; also letters of regret for non-attendance from Dr. R. R. Hammond and others. Under the head of new business it was moved by G. A. Jonson and seconded by S. H. Johnston that the Secretary be instructed to correspond with the Secretary of the North Eastern Iowa Association upon the idea of holding a joint meeting of the two Associations at Ames, Iowa, some time during the summer. The motion was carried. Dr. Gibson then suggested that the Association take some action looking to the bettering of the standard of breeding stock in Iowa, and the Doctor further suggested that the members of the Association and all veterinarians might promote the cause by writing articles for their local papers and the stock journals. It was then moved and seconded that the Association recommend a law bearing upon this subject. This motion brought out a spirited discussion and considerable difference of opinion as to the advisabilty of such a step.

Professor Niles then presented some notes on experiments upon the use of Pilocarpin, also on the different disinfectants in general use, as applied to wounds, which elicited an interesting discussion. It was then moved and seconded that the President declare Dr. W. H. Heck a member of the Association, which he did, expressing his pleasure in so doing. Dr. G. A. Jonson then presented a paper on "Difficult Parturition and the After Treatment," which was followed by an interesting discussion, after which the meeting adjourned to meet at the call of the Secretary. L. U. SHIPLEY, *Secretary.*

